# Miscellaneous

**Published:** 1890-11

**Authors:** 


### MISCELLANEOUS.

### BORN WITH A CHILL.

A traveler, riding through the Muscadine neighborhood, came upon a man sitting on a log near the roadside. He was sallow and lean, with sharp knob cheek bones, and with hair like soiled cotton. The day was intensely hot, but he sat in the sun, although near him a tangled grape vine cast a most inviting shade.

"Good morning," said the traveler, reining up.

"Hi."

"You live here, I suppose?"

"Jest about."

"Why don't you sit over there in the shade?"

"Will when the time comes."

"What do you mean by when the time comes?"

"When the fever comes on."

"Having chills, are you?"

"Sorter."

"How long have you had them?"

"Forty-odd year."

"How old are you?"

"Forty odd year."

"Been shaking all your life, eh?"

"Only half my life; fever the other half."

"Why don't you move away from here?"

"Becase I'm afeered I might not have good health nowhar else."

"Gracious alive, do you mean to say that having chills all the time is good health?"

"Well, health mout be wuss. Don't b'lieve in swappin' off suthin' that I'm used to fur suthin' I don't know nothin' about. Old fashioned, every-day chills air good enough for me. Some folks, when they git a little up in the world, mout want to put on airs with dyspepsia and bronckichus, and glanders and catarrh, but, as I 'lowed to my wife, old chills and fever war high enough for us yit awhile. A chill may have drawbacks, but it has its enjoyments, too."

"I don't see how a chill can be enjoyable."

"Jest owin' to how you air raised. When I have a chill it does me a power of good to stretch, and I tell you that a fust-rate stretch when a feller is in the humor ain't to be sneezed at. High-o-hoo!" He gaped, threw out his legs, threw back his arms and stretched himself across the log. "It's sorter like the itch," he went on. "The itch has its drawbacks, but what a power of good it does a man to scratch. Wall, my fever is comin' on now, and I reckon I'll hurry and git up thar under the shade."

He moved into the shade and stretched himself again.

"How long will your fever last?"

"Wall, I don't know, exackly ; three hours, mebby."

"Then what ?"

"Well, I'll funter around awhile, chop up a little wood to get a bite to eat with, swap a hoss with some feller, mebby, and then fix myself for another chill."

"Have you much a family ?"

"Wife and grown son. He's about the ablest chiller in the country ; w'y, when he's got a rale good chill on he can take hold of a tree and shake off green persimmons. Wall, have you got to go ?"

"Yes."

"Good-by, then. When you get tired livin' up thar among them new-fangled diseases come down here whar everything is old fashioned, comfortable and honest."

---

Heartburn may be relieved almost instantly if half a teaspoonful of table salt be dissolved in a wineglassful of cold water and then drank.

---

Tincture of camphor or tincture of myrrh added in the proportion of ten or twelve drops to a glass of water, is good for rinsing the mouth mornings.

---

Do not allow a patient with colic to suffer until the physician arrives, but give a large injection of warm water ; two quarts with tincture of opium, for an adult, and less quantity for a child.

---

Quinine is robbed of its bitter taste by combining it with sugar of milk and some bicarbonate of soda. Capsicum, ginger, or other aromatics, are also used in combination with quinine to prevent its disagreeable head symptoms and for other valuable improvement in its administration.

---

Macaroni.—Macaroni is such a cheap, palatable, healthful and easily prepared food that I wonder it is not more generally used. It can be prepared in so many forms, that although on the table often one will not tire of it. A pound package only costs twelve cents, and half of that amount will be sufficient for a family of six persons. Break the macaroni into inch-long pieces. Have about three pints of water in a sauce-pan, and when it is boiling throw in the macaroni, and add a tablespoonful of salt, cover the sauce-pan and let it boil about twenty minutes, then turn it into a colander to drain. Put a teaspoonful of butter and half a teacupful of cream or milk into the sauce-pan, and set it on the stove ; wet half a teaspoonful of flour, or better, of corn starch, with a little milk, pour it into a sauce-pan, and when it boils, put the macaroni back into the sauce-pan, season with a little cayenne or black pepper, stir well, and serve as soon as hot. This is plain boiled macaroni. If cheese is liked, add half a teacupful of cheese after putting it back in the sauce-pan, and stir well together. Another time, after boiling and draining the macaroni, put it in a buttered pudding dish, and pour over it a pint of stewed tomato, seasoned as if for the table, sprinkle bread crumbs over the top, and set it in the oven until slightly browned over the top. Another way is to boil the macaroni in soup stock, or the broth in which meat has been boiled.

### DRUGGED BAKING POWDERS.

### HOW THE PRESENCE OF AMMONIA IN A POWDER MAY BE DETECTED.

Ammonia and alum are the most common adulterants used in the manufacture of baking powders. The Government report shows that a large percentage of the baking powders on the market contain either one or the other, or both these pernicious drugs. Ammonia particularly is in very general use. So great has the demand for it increased with the increased use of the baking powders, that an ammonia trust has been formed and the price of the drug has gone up. Such baking powder companies as are not share holders in the trust company have suffered a material decrease in profits as a consequence.

This wholesale use, in an article of daily food, of one of the most insidious and injurious poisons is simply criminal. Slow ammonia poisoning produces diseases of the stomach and is particularly injurious to the complexion. The presence of ammonia in a baking powder, however, can be very easily detected. Mix one heaping teaspoonful of baking powder with one teaspoonful of water in a tin cup ; boil thoroughly for a few moments, stir to prevent burning, and if ammonia is present you can smell it in the rising steam. As baking powder, when first thrown into water, will effervesce, care should be taken to not mistake bubbling for boiling. When a baking powder has not a list of its ingredients printed on its label this test should always be applied.

Ammonia, in a measure, takes the place of the expensive ingredients of baking powders. Where sales are large, the profits which this adulteration yields are enormous, and some of the best known manufacturing firms whose standing would seem a guarantee against fraud are engaged in it. Even some of those concerns which advertise the most extensively, and whose advertisements ring with the "purity" of the article, are among the worst offenders.

---

Boils.—Boils are caused by germs, but it is not fully determined just how the germs find access. They are probably received through some slight abrasion or other injury to the skin. The pus which is discharged is full of germs. A slight injury in the vicinity of any of the glands of the body through which germs are given opportunity for entrance, is often followed by enlargement of the glands. An injury to the face, for instance, will cause the glands of the neck and jaw to swell. Sometimes from inflamed tonsils, germs find access, and are carried by the lymphatics along to some point where they obstruct the blood vessels and form a tiny clot which gives the germs a chance to feed and grow upon dead blood, and in this way develop a boil. Sometimes germs of consumption are similarly taken to the lungs. Enlarged lymphatic glands about the neck or elsewhere should receive attention, because they may lead to something else. They often take on tuberculosis degeneration, and after a while reach the lungs. It is best to have the glands removed by surgery while the enlargement is still slight. Otherwise gland below gland may be found affected, and the operation come to be quite a serious one. It is very rare that they can be cured by any remedy. A simple enlargement of the glands can sometimes be driven away by arsenic, but may develop a disorder worse than the enlarged glands.

FOR DYSPEPSIA, USE HORSFORD'S ACID PHOSPHATE.—Dr. Lorenzo Waite, Pittsfield, Mass., says : "From its use, for a period of about eight weeks, to the exclusion of all other remedies, I attribute the restoration to health of a patient who was emaciated to the last degree, in consequence of nervous prostration and dyspepsia. This patient's stomach was in such an irritable condition that he could not bear either liquid or solid food. An accomplished physician of many years' experience, whom I called in consultation, pronounced his case an incurable one. At this stage I decided to use Horsford's Acid Phosphate, which resulted as above mentioned."

ZADOC PORTER's remedies still remain at the head for all coughs and stomach troubles ; see advertisement of Ruckel & Hendel, who are the proprietors of these medicines.

THE NEW YORK CANCER HOSPITAL has made an appeal for financial aid in carrying out the humane purposes of its organization. In 1889 it treated 131 free patients, on an average stay of forty days. None were refused admittance on account of their inability to pay. Such a charity deserves well of the unafflicted and well to do.

THE NEW YORK PASTEUR INSTITUTE, located at 178 W. 10th street, makes an excellent showing. It was opened for patients last February, and up to the 15th of October, 610 patients had been treated. Of these 130 had unquestionably been bitten by animals afflicted with rabies, and to-day all are alive and doing well. The officers are Paul Gibier, M. D., Director, C. Van Schaick, M. D., Asst., A. Liautard, M. D., M. V., Consulting Veterinarian.

### DESCRIPTION OF THE HASSEN TOWER IN AFRICA.

The Sma-Hassen Tower at Rabat, Africa, is a superb structure, and although in parts unfinished and damaged by lightning, is still lordly and beautiful. Built of hewn stone brought from Spain and by the hands of Christian captives, and 180 feet from base to summit, it presents on the outside three tiers of large and elegant arches over comparatively small windows, and above the topmost arch a deep honeycomb of exquisite carving. It has a simple grandeur of proportion that is peculiarly its own and very impressive, says an exchange. The ascent of the tower is made not by stairs, but by a series of inclined planes, up which a horse might be ridden three abreast, as Leo Africanus asserts.

The lowest of these inclined planes, which are made of a concrete of lime and sand, very hard and durable, was broken away in the time of the Emperor Sidi Mahomet, and by his order, so that now a ladder has to be used before a footing can be got. As the ascent is made a number of spacious chambers, chill, solemn and tenantless, except by owls and bats, are passed, and when the top is reached a magnificent view is obtained of the restless Atlantic. The tower is not merely a stately sentinel of the great mosque, but a lookout station and a beacon for ships at sea.

---

### CONSOLATION.

**By Rossiter W. Raymond.**

Beside the dead I knelt for prayer,
And felt a presence as I prayed—
Lo ! it was Jesus standing there ;
He smiled : "Be not afraid !"

"Lord, Thou hast conquered death, we know ;
Restore again to life," I said—
"This one who died an hour ago."
He smiled : "She is not dead !"

"Asleep, then, as Thyself did say,
Yet Thou canst lift the lids that keep
Her prisoned eyes from ours away."
He smiled : "She does not sleep !"

"Nay, then, though haply she do sleep,
And look upon some fairer dawn,
Restore her to our hearts that ache."
He smiled : "She is not gone !"

"Alas ! we know too well our loss,
Nor hope again one joy to touch
Until the stream of death we cross."
He smiled : "There is no such !"

